# Long-acting reversible contraceptives utilization and its determinants among married Yemeni women of childbearing age who no longer want children

**DOI:** 10.1097/MD.0000000000030717

**Published:** 2022-10-07

**Authors:** Michael Boah, Abdul-Nasir Issah, Isaac Demuyakor, Dalia Hyzam

**Affiliations:** a Department of Epidemiology, Biostatistics, and Disease Control, School of Public Health, University for Development Studies, Tamale, Ghana; b Department of Health Services, Policy, Planning, Management and Economics, School of Public Health, University for Development Studies, Tamale, Ghana; c Department of Health Policy and Hospital Management, School of Health Management, Harbin Medical University, China; d Women’s Center for Research and Training, The University of Aden, Yemen.

**Keywords:** contraception, family planning, reproductive women, Yemen

## Abstract

Some contraceptive methods, such as long-acting and permanent methods, are more effective than others in preventing conception and are key predictors of fertility in a community. This study aimed to determine which factors were linked to married women of childbearing age who no longer desired children using long-acting reversible contraceptives (LARCs) in Yemen. We used a population-based secondary dataset from Yemen’s National Health and Demographic Survey (YNHDS), conducted in 2013. The study analyzed a weighted sample of 5149 currently married women aged 15 to 49 years who had no plans to have children. Logistic regression analyses were used to investigate the parameters linked to the present use of LARCs. The final model’s specifications were evaluated using a goodness-of-fit test. An alpha threshold of 5% was used to determine statistical significance. Of the total sample, 45.3% (95% CI: 43.3–47.4) were using contraception. LARCs were used by 21.8% (95% CI: 19.6–24.1) of current contraceptive users, with the majority (63.8%) opting for short-acting reversible contraceptives (SARCs). In the adjusted analysis, maternal education, husbands’ fertility intention, place of residence, governorate, and wealth groups were all linked to the usage of LARCs. According to the findings, women whose spouses sought more children, for example, were more likely to use LARCs than those who shared their partners’ fertility intentions (AOR = 1.44; 95% CI: 1.07–1.94; *P* = .015). In this study, married women of reproductive age who had no intention of having children infrequently used contraception and long-acting methods. Improving women’s education and socioeconomic status could contribute to increasing their use of LARCs.

## 1. Introduction

Contraception helps couples and individuals achieve their reproductive goals and exercise their rights to choose whether to have children. Globally, the decline in fertility rate from 3.2 live births per woman in 1990 to 2.5 in 2019 indicates that women have fewer children today than three decades ago.^[1]^ The fall in the global fertility rate coincided with an increase in access to and use of contraception among reproductive women, as well as a decline in the global rate of unwanted pregnancies.^[1,2]^

The importance of contraceptive use to progress in all major sustainable development goal themes is reflected in sustainable development goal indicator 3.7.1, the proportion of women of reproductive age whose family planning (FP) needs are met with modern contraceptives.^[1]^ In recent years, the number of contraceptive methods available to women has increased. However, some women who want to avoid pregnancy do not use any method of contraception due to lack of access, inadequate knowledge, side effects, infrequent or no sex, sub-fecundity, and partner opposition.^[3]^ Furthermore, some existing contraceptive methods are imperfect or user-dependent, and some women use them incorrectly or experience method failures.^[4,5]^ For programming purposes, contraceptive methods are classified as long-acting reversible contraceptives (LARCs), permanent contraceptive methods (PCMs), and short-acting reversible contraceptives (SARCs). Although SARCs, like LARCs, are extremely effective methods of birth control, in typical use, they leave room for error and have much lower efficacy in preventing unintended pregnancies.^[5]^ On the other hand, LARCs, are safer and more effective and are associated with very low failure rates, making them the ideal form of contraception for married women who want to limit childbearing.^[4,6–8]^ Regrettably, the use of LARCs by women of reproductive age in many settings worldwide has been low.^[9]^

Early marriages are common in Yemen, one of the poorest countries in the Middle East and North Africa region. Previous reports have claimed that early marriage and a low level of maternal education contribute significantly to the country’s high fertility rate.^[10]^ However, according to the Yemen National Health and Demographic Survey (YNHDS), the country’s fertility rate decreased from 6.5 live births in 1997 to 4.4 in 2013, with increased access to and use of contraception, especially modern techniques, likely contributing to these trends.^[11]^ The relationship between contraceptive use and birth rate is also influenced by the methods used by women, such as long-acting and permanent methods, which are more effective than others in preventing pregnancy. Existing studies demonstrate that the use of LARCs by married women of reproductive age who do not want to have children has received little attention in Yemen.^[11–13]^

Our literature search yielded studies that investigated the use of LARCs among reproductive women in sub-Saharan Africa (SSA)^[14,15]^ and Nepal.^[16]^ Nonetheless, the emphasis, study sample, or geographical location of these related studies limits their generalizability to the study setting. For example, in SSA, injectables are the most commonly used method by women of reproductive age, whereas permanent and long-acting methods are more common in Asia, indicating that women’s contraception methods vary depending on their geographical location.^[9]^ The Nepalese study, on the other hand, was conducted on ever-married women whose fertility intentions were unknown. However, it has been established that desire for children is a powerful predictor of contraceptive behavior.^[16–19]^

This current study sought to examine the use of LARCs and its determinants among married women with no intention of having children in Yemen, where early marriage is more common and births are more closely spaced.

## 2. Methods

### 2.1. Theoretical framework

This study modified Andersen and Newman behavioral model of health service use, which has been widely utilized in research on the use of health services, including family planning.^[20,21]^ The model illustrates how predisposing circumstances (also called psychosocial factors) influence one’s utilization of health care. In other words, four domains – attitudes, knowledge, social norms, and perceived control – influence the decision-making process related to an individual’s planned behavior. Enabling variables are those that make it easier to use the service, such as the availability of adequate individual and community-level resources. Overall, one’s access to and ability to pay for healthcare services may limit their utilization. The term “need” refers to how people describe their health and functional state, which can be negatively or positively influenced depending on how bad their health is. So, the researchers hypothesized that in the study environment, predisposing, enabling, and need factors influence the use of LARCs by married women of reproductive age.^[22]^

### 2.2. Study design

This study had a cross-sectional design. The researchers used a secondary dataset from the 2013 YNHDS.

### 2.3. Data

This study relied on data from the 2013 YNHDS, which was implemented by the Ministry of Public Health and Population in collaboration with the Central Statistical Organization. The sample for the original survey was selected from 213 clusters in urban areas and 587 clusters in rural areas, giving a total of 800 clusters. The sampling frame used was taken from the 2004 General Population Housing and Establishment Census. Of the 19,517 households selected for inclusion, 18,027 were included in the study. The women’s file (dataset) was used in this study. The dataset contains information about women’s background characteristics such as age, education, type of place of residence, governorate, wealth quintile, and reproductive health data such as fertility and fertility preferences, as well as knowledge and use of FP methods. Information about how the 2013 survey was conducted, including the questionnaire that was used to collect data, is contained in the final report.^[11]^

### 2.4. Sample size

The original survey interviewed 25,434 women of reproductive age (15–49 year). There were 15,649 married people among those who participated in the study. The researchers eliminated 2166 pregnant women from the sample, leaving 13,483 non-pregnant women in the study. The current study focused on the use of LARCs by married women who no longer wanted to have children. Consequently, the research was limited to 6209 women who said they no longer wanted children to meet the study’s objectives. Records with missing data for any of the study’s explanatory factors (n = 1157) were also eliminated. The final sample of women in this study was made up of 5052 (weighted N = 5149) married women of childbearing age who were not pregnant.

### 2.5. Study variables

#### 2.5.1. Dependent variables.

In this study, two outcome factors were investigated. The first step was to estimate the percentage of married reproductive women in the study population who were currently using any type of contraception. This was done to provide an estimate of contraceptive use among married women of reproductive age who no longer wanted children in the study setting. Current contraceptive use is a binary dummy variable, with “0” denoting non-users and “1” denoting current users. Participants’ self-reports of contraceptive use by themselves or their husbands provided this information. The current usage of LARCs, the second outcome variable,is also a binary variable coded as “0” for non-users and “1” for current users. Only participants who reported using any method of contraception at the time of data collection were classified in this manner; non-contraceptive users, in other words, were not included in this analysis. According to the information available in the dataset on the contraceptive methods used as reported by the participants, the contraceptive methods were classified as LARCs, which included intrauterine devices, implants, and norplant; SARCs, which included pills, injections, diaphragms, male and female condoms, lactational amenorrhea method, and other modern methods; PCMs, which included male and female sterilization; and lastly, traditional methods (TMs), which included periodic abstinence, withdrawal method, and other traditional methods.

#### 2.5.2. Independent variables.

The researchers selected specific variables for inclusion in the study as potential determinants based on the current literature and variables accessible in the dataset. The predisposing factors in this study included maternal age, age at first marriage, maternal employment status, maternal educational level, maternal decision-making autonomy regarding health, and number of living children. The enabling factors were husband’s educational level, husband’s employment status, place of residence, governorate, wealth, media exposure to FP information (print media, audio, and audiovisual), and interaction with the health care system (whether the woman visited the health facility or was visited by a health worker in the last 12 mo). Need factors included the husband’s desire for children. Most variables were utilized exactly as they were in the demographic and health survey (DHS) dataset, including maternal age, place of residence, governorate, and wealth quintile (as a composite variable). Based on the existing DHS dataset, new variables were created, such as the number of living children and the employment of the woman and her husband/partner.

### 2.6. Data analysis

STATA/IC 15.0 (StataCorp LLC, College Station, TX) was used to analyze the data. To account for the sampling design used by the DHS, weights were applied to the data to generate nationally representative statistics.^[23]^ Descriptive statistics were used to report the distribution of the population analyzed by key characteristics, including sociodemographic and economic factors. A Pearson design-based Chi-square (*χ*^2^) test was used to assess differences between current LARCs users and non-users. Binary logistic regression was used to model the factors associated with the dichotomous dependent variable, current use of LARCs. All independent variables were forced into the model to assess their independent association with current use of LARCs. The final model for this study was built after controlling for the confounding factors. The specifications of the final model was evaluated using the “goodness-of-fit test” developed by Archer and Lemeshow for logistic regression models fitted with survey data.^[24]^ There was no statistical evidence to ascribe a lack of fit to the final model, as evidenced by the probability value (*P* = .986). Statistical significance was set at a probability value (*P* value) of not more than .05.

### 2.7. Ethical considerations

The original survey was approved by the Institutional Review Board of the Inner-City Fund International and ORC Macro. Before the interview, all respondents were provided information about the survey and agreed to participate by submitting written informed consent. The current study was a secondary analysis; therefore, approval by an institutional review board was not required. Permission to use the data for the current study was obtained from the DHS program.

## 3. Results

### 3.1. Background characteristics of the participants

The background characteristics of the participants are shown in Table 1. The majority of women (65.5%) had no formal education, were unemployed (88.2%), resided in rural areas (69.6%), were married before the age of 18 (62.1%), had at least 5 living children (62.7%), and were active in health-related decision-making (55.9%), according to the data. Regarding recent exposure to family planning information, 28.6% said they heard it on the radio, 46.0% said they saw it on television, and 8.8% said they read it in newspapers or magazines. Approximately 5% of the women said they were visited by a family planning worker in the preceding year, whereas 43.0% went to a health facility (Table 1).

**Table 1:** The background characteristics of the study sample (Weighted N = 5, 149).

Variable	Number	Percent
Age group (yr)		
15–19	57	1.1
20–24	381	7.4
25–29	826	16.0
30–34	1007	19.6
35–39	1192	23.2
40–44	918	17.8
45–49	768	14.9
Educational level of woman		
No education	3373	65.5
Fundamental	1324	25.7
At least Secondary	452	8.8
Educational level of partner		
No education	1560	30.3
Fundamental	1892	36.7
At least secondary	1697	33.0
Employment of partner		
Unemployed	191	3.7
Employed	4958	96.3
Employment of woman		
Unemployed	4541	88.2
Employed	608	11.8
Type of place of residence		
Urban	1563	30.4
Rural	3586	69.6
Governorate		
Ibb	500	9.7
Abyan	90	1.7
Sanaa city	459	8.9
Al-baidha	251	4.9
Taiz	699	13.6
Al-jawf	37	0.7
Hajjah	393	7.6
Al-hodiedah	682	13.2
Hadramout	149	2.9
Dhamar	342	6.6
Shabwah	70	1.4
Sadah	178	3.5
Sanaa	304	5.9
Aden	178	3.5
Lahj	166	3.2
Mareb	29	0.6
Al-mhweit	162	3.1
Al-mhrah	13	0.3
Amran	203	3.9
Aldhalae	134	2.6
Reimah	110	2.1
Age at first marriage		
<18 yr	3196	62.1
18 yr	1953	37.9
Number of living children		
0	18	0.3
1	144	2.8
2	408	7.9
3	595	11.6
4	754	14.6
5 or more children	3230	62.7
Decision-making on woman’s health		
Not involved	2271	44.1
Involved	2878	55.9
Husband’s desire for children	5149	
Both want same	1901	36.9
Husband wants more	2092	40.6
Husband wants fewer	347	6.7
Don’t know	809	15.7
Wealth quintile		
Poorest	1076	20.9
Poorer	1010	19.6
Middle	1063	20.6
Richer	964	18.7
Richest	1036	20.1
Heard of FP on radio last few months		
No	3676	71.4
Yes	1473	28.6
Heard of FP on TV last few months		
No	2783	54.0
Yes	2366	46.0
Heard of FP in newspaper/magazine last few months		
No	4695	91.2
Yes	454	8.8
Visited by FP worker last 12 mo		
No	4901	95.2
Yes	248	4.8
Visited health facility last 12 mo		
No	2935	57.0
Yes	2214	43.0

FP = family planning, TV = television.

### 3.2. Current use of contraception among married, non-pregnant women who no longer want children

Of the total sample analyzed (N = 5149), 2815 women reported not currently using any method of contraception, while 2334 reported currently using a contraceptive method, yielding a prevalence of 45.3% (95% CI: 43.3–47.4) for the current use of any form of contraception among married, non-pregnant women of childbearing age who no longer wanted children in the study area.

### 3.3. Current LARCs use among married non-pregnant women who were on contraception

**Figure 1.**
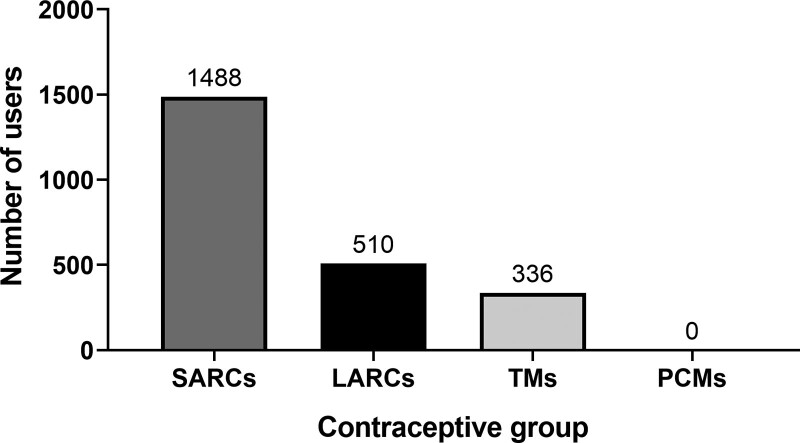
Current use of long-acting reversible contraceptives (LARCs) among current contraceptive users (n = 2334). PCMs = permanent contraceptive methods, SARC = short-acting reversible contraceptives, TMs = traditional methods.

We built a new model to estimate the percentage of married women who used LARCs. In this model, we only examined married women who said they were currently using contraception (n = 2334). The literature, which revealed that some methods, such as long-acting methods, had lower failure rates than others, informed the modeling. The results indicated that 1488 (63.8; 95%: 61.1–66.3) current contraceptive users were using SARCs, 510 (21.8%; 95% CI: 19.6–24.1) were using LARCs, and 336 (14.4%; 95% CI: 12.7–16.3) were using TMs. The PCMs were not used by anyone (Fig. 1).

There were several notable differences between present LARC users and non-users. On the one hand, the current users and non-users were similar in terms of age, age at first marriage, employment of women and husbands, women’s decision-making autonomy, husband’s desire for children, and across the indicators assessing women’s contact with the health care system. However, they differed considerably from non-users in terms of the women and husbands’ educational levels, place of residence, governorate, number of living children, wealth quintile, and all indicators of mass media exposure to FP information (Table 2).

**Table 2:** Long-acting reversible contraceptives use by background characteristics of participants (Weighted N = 2334).

Variable	Weighted percentage		*P* value
	Not using (n = 1824)	Using (n = 510)	
Age group (yr)			.252
15–19	87.0	13.0	
20–24	85.0	15.0	
25–29	78.6	21.4	
30–34	73.8	26.2	
35–39	77.6	22.4	
40–44	79.4	20.6	
45–49	81.4	18.6	
Educational level of woman			<.001
No education	83.2	16.8	
Fundamental	73.5	26.5	
At least secondary	69.8	30.2	
Educational level of partner			<.001
No education	84.1	15.9	
Fundamental	81.0	19.0	
At least secondary	73.3	26.7	
Employment of partner			.538
Unemployed	81.4	18.6	
Employed	78.1	21.9	
Employment of woman			.297
Unemployed	78.5	21.5	
Employed	75.6	24.4	
Type of place of residence			<.001
Urban	70.7	29.3	
Rural	83.4	16.6	
Governorate			<.001
Ibb	72.8	27.2	
Abyan	76.7	23.3	
Sanaa city	62.1	37.9	
al-baidha	78.7	21.3	
Taiz	84.3	15.7	
Al-jawf	79.5	20.5	
Hajjah	88.0	12.0	
Al-hodiedah	78.4	21.6	
Hadramout	85.4	14.6	
Dhamar	78.7	21.3	
Shabwah	84.6	15.4	
Sadah	93.8	6.2	
Sanaa	67.1	32.9	
Aden	82.1	17.9	
Lahj	91.3	8.7	
Mareb	75.4	24.6	
Al-mhweit	85.6	14.4	
Al-mhrah	100.0	0.0	
Amran	86.7	13.3	
Aldhalae	81.8	18.2	
Reimah	83.1	16.9	
Age at first marriage			.138
<18 yr	76.7	23.3	
18 yr	80.3	19.7	
Number of living children			.009
0	100.0	0.0	
1	90.8	9.2	
2	78.5	21.5	
3	79.7	20.3	
4	70.1	29.9	
5 or more children	79.4	20.6	
Decision-making on woman’s health			.690
Not involved	77.6	22.4	
Involved	78.5	21.5	
Husband’s desire for children			.095
Both want same	81.1	18.9	
Husband wants more	74.8	25.2	
Husband wants fewer	80.0	20.0	
Don’t know	77.7	22.3	
Wealth quintile			<.001
Poorest	94.0	6.0	
Poorer	82.8	17.2	
Middle	81.2	18.8	
Richer	72.8	27.2	
Richest	72.0	28.0	
Heard of FP on radio last few months			.036
No	79.6	20.4	
Yes	74.8	25.2	
Heard of FP on TV last few months			.020
No	81.2	18.8	
Yes	75.8	24.2	
Heard of FP in newspaper/magazine last few months			.041
No	79.1	20.9	
Yes	71.5	28.5	
Visited by FP worker last 12 mo			.720
No	78.2	21.8	
Yes	76.5	23.5	
Visited health facility last 12 mo			.419
No	79.1	20.9	
Yes	77.2	22.8	

FP = family planning, TV = television.

### 3.4. Independent factors associated with the current use of LARCs among current contraceptive users who no longer want children

A design-based multiple logistic regression model was employed to identify the determinants of current use of LARCs among current contraceptive users in the study. The results showed that factors, including maternal educational level, type of place of residence, governorate, husband’s desire for children, and wealth quintile, demonstrated a statistically significant association with the use of LARCs among the study population. As shown in Table 3, married women with fundamental or at least secondary education had increased odds of reporting the current use of LARCs compared to their counterparts with no formal education (AOR = 1.54; 95% CI: 1.09–2.17 and 1.88; 95% CI: 1.11–3.16, respectively). Similarly, higher odds of LARC use were noted among women whose husbands desired more children compared to women who desired the same number of children as their husbands (AOR = 1.44; 95% CI: 1.07–1.94). In contrast, women from rural settings reported lower odds of LARC use compared to urban residents (AOR = 0.54; 95% CI: 0.34–0.85). Women in Taiz, Hajjah, Hadramout, Sadah, Aden, Lahj, Al-mhweit, and Amran had lower odds of reporting the use of LARCs than those in Ibb (Table 3).

**Table 3:** Factors associated with the current use of long-acting reversible contraceptives among women who no longer want children (Weighted N = 2334).

Variable	AOR(95% CI)	*P* value
Age group (yr)		
15–19 (Ref)	1.00	
20–24	1.66 (0.25–11.20)	.600
25–29	2.98 (0.42–21.18)	.274
30–34	4.20 (0.58–30.30)	.154
35–39	4.03 (0.55–29.60)	.171
40–44	4.23 (0.53–33.53)	.172
45–49	3.97 (0.55–28.59)	.170
Educational level of woman		
No education (Ref)	1.00	
Fundamental	1.54 (1.09–2.17)	.014
At least Secondary	1.88 (1.11–3.16)	.018
Educational level of partner		
No education (Ref)	1.00	
Fundamental	1.04 (0.69–1.58)	.836
At least Secondary	1.35 (0.86–2.10)	.190
Employment of partner		
Unemployed (Ref)	1.00	
Employed	0.84 (0.40–1.76)	.650
Employment of woman		
Unemployed (Ref)	1.00	
Employed	1.14 (0.79–1.64)	.487
Type of place of residence		
Urban (Ref)	1.00	
Rural	0.54 (0.34–0.85)	.009
Governorate		
Ibb (Ref)	1.00	
Abyan	0.66 (0.28–1.56)	.345
Sanaa city	0.95 (0.55–1.63)	.847
Al-baidha	0.58 (0.24–1.38)	.217
Taiz	0.44 (0.24–0.81)	.009
Al-jawf	0.53 (0.24–1.15)	.108
Hajjah	0.39 (0.19–0.81)	.011
Al-hodiedah	0.52 (0.25–1.06)	.071
Hadramout	0.32 (0.15–0.66)	.002
Dhamar	0.90 (0.43–1.87)	.783
Shabwah	0.42 (0.15–1.16)	.095
Sadah	0.17 (0.07–0.42)	<.001
Sanaa	1.45 (0.80–2.62)	.223
Aden	0.33 (0.18–0.59)	<.001
Lahj	0.26 (0.11–0.59)	.001
Mareb	0.83 (0.41–1.66)	.599
Al-mhweit	0.46 (0.22–0.96)	.038
Al-mhrah^*^	-	
Amran	0.50 (0.27–0.94)	.032
Aldhalae	0.58 (0.28–1.23)	.157
Reimah	0.78 (0.27–2.22)	.640
Age at first marriage		
<18 yr	1.00	
≥ 18 yr	0.75 (0.55–1.03)	.076
Number of living children		
0–4 (Ref)	1.00	
>4	0.82 (0.58–1.15)	.257
Decision- making on woman’s health		.672
Not involved (Ref)	1.00	
Involved	0.88 (0.67–1.15)	.344
Husband’s desire for children		
Both want same (Ref)	1.00	
Husband wants more	1.44 (1.07–1.94)	.015
Husband wants fewer	1.12 (0.63–1.97)	.706
Don’t know	1.31 (0.81–2.12)	.277
Wealth quintile		
Poorest (Ref)	1.00	
Poorer	3.01 (1.57–5.78)	.001
Middle	2.98 (1.54–5.77)	.001
Richer	3.23 (1.59–6.56)	.001
Richest	2.30 (1.03–5.11)	.042
Heard of FP on radio last few months		
No (Ref)	1.00	
Yes	1.01 (0.75–1.35)	.961
Heard of FP on TV last few months		
No (Ref)	1.00	
Yes	0.98 (0.73–1.32)	.896
Heard of FP in newspaper/magazine last few months		
No (Ref)	1.00	
Yes	0.96 (0.61–1.51)	.872
Visited by FP worker last 12 mo		
No (Ref)	1.00	
Yes	1.28 (0.73–2.25)	.395
Visited health facility last 12 mo		
No (Ref)	1.00	
Yes	1.10 (0.83–1.47)	.493

Model fitness test results: *F*-adjusted test statistic = *F* (9, 588) = 0.253; Prob > *F* = 0.986.

AOR = adjusted odds ratio, FP = family planning, Ref = Reference group, TV = television.

* Output was empty for this governorate due to 0 users of LARCs (see Table 2 for more information).

## 4. Discussion

If married couples no longer want children, LARCs and PCMs are the best solutions for stopping childbearing.^[5]^ The goal of this study was to use nationally representative data to estimate LARCs use and its determinants among married women of reproductive age with no fertility ambitions in Yemen. Less than half of the married women who did not wish to have children in Yemen used contraception to limit childbearing, indicating a major unmet need for family planning. The use of SARCs was reported by the majority of current contraceptive users, but LARCs was reported by roughly one in five married women. The low use of contraception among married women with no intention of having children is not unique to the current study’s setting. It has been documented that only about a third of women with no fertility intentions in SSA use modern contraception to space or limit child bearing.^[14]^ Population-based studies have estimated that the use of LARCs among married women ranged from 1.9% to 55.0% in Africa.^[15,25]^ A nationwide study reported that approximately 5% of currently married Nepalese women of reproductive age use LARCs.^[16]^ The evidence shows that the use of LARCs reported in the current study is higher than that reported by the Nepalese study but within the range reported in SSA, which could be attributed to differences in the study population as well as access to health services. For example, unlike the Nepalese study, the current study examined currently married reproductive women who had no plans to have children. Indeed, it has been recognized that the desire for children is a known predictor of the use of contraception to limit childbearing.^[26–28]^

It is worth mentioning that, in this study, more than 8 out of 10 women with no fertility intentions used SARCs and TMs to limit childbearing. This evidence has negative implications for controlling the rapid population growth in the study setting. This is because these methods are associated with high failure rates, particularly for TMs users. The literature explains that more than six out of every 100 pill users and more than eight out of every 100 condom users become pregnant within the first year of use.^[4]^ The resulting unintended pregnancies can have serious consequences for women and their families. Myths and misconceptions about LARCs’ use, such as risk of cancer, genital tract infections, altered sexual function, and delayed conception following discontinuation, contribute to negative attitudes toward LARCs.^[29,30]^

After controlling for confounding factors, the study identified various predisposing, enabling, and need factors, that were significantly associated with the use of LARCs among married women with no desire for children in the study setting. The study showed that women with some level of formal education were more likely to report the current use of LARCs than those with no formal education. Other studies have also established that higher maternal education is associated with increased use of LARCs among women of reproductive age.^[15,16,31]^ In Southeast Asia, low levels of education among women have been cited as a major impediment to the adoption of long-term contraceptive methods.^[32]^ In a broader context, causal links have been drawn between higher maternal education and the use of health services in many countries.^[33]^ Education yields numerous benefits, including enhanced access to health information and decision-making power to act on that knowledge. Furthermore, well-educated women are more likely to live in cities and wealthy households, which improves their access to health care and their capacity to handle any financial hurdles connected with getting LARCs.^[34,35]^

Various factors influence women’s decisions to use contraception. While some women have complete control over their reproductive goals, others may face a complex web of influence. Nevertheless, intention and knowledge are two critical components of the decision-making process.^[36]^ According to the findings of this study, women whose husbands desired more children were more likely to use LARCs than were those who shared their partners’ fertility intentions. Given the study’s sample, it is plausible to assume that women are surreptitiously using LARCs to fulfill their reproductive objectives. Women with anti-family planning spouses use LARCs, particularly implants, covertly to regulate their reproductive desires.^[37]^ Importantly, recent research from Asia shows that when couples dispute future fertility plans, the wife’s choice about having children is more closely linked to her contraceptive behavior.^[19]^

It appears that findings on the association between fertility intentions and the use of LARCs are inconclusive. Women in SSA who no longer desire children are reportedly more likely to use LARCs than those who desire additional children.^[15]^ In contrast, according to a study conducted in Asia, women who desired another child in the future were more likely to use LARCs than those who did not.^[16]^ Despite the contradictory nature of these findings, convergence is the premise for the utilization of LARCs. Women are interested in LARCs to limit or space their childbearing.^[38,39]^

The study discovered that the use of LARCs was differentially distributed between urban and rural women and by governorate, which we attribute to the dissimilarities in socio-economic and political conditions as well as access to health services.^[12]^ A study in Ethiopia found that the region of residence was associated with the use of LARCs among reproductive women; women in developed regions had a greater likelihood of using LARCs than their counterparts.^[25]^ Also, most researchers agree that rural women have limited access to and utilization of reproductive health services, including contraceptives.^[32,40]^ National data from Yemen indicates that more than half of women of reproductive age rely on the public sector for contraceptive needs.^[11]^ Unfortunately, the country’s protracted conflict has deteriorated the already fragile health system, resulting in severe gaps in the availability and access to vital health treatments, including family planning, among urban and rural women. Direct attacks on Yemen’s public health institutions have resulted in a shortage of personnel and supplies, as well as a reduction in overall functionality.^[41,42]^ It has also been found that the damage to health facilities varied by governorate, with Aden, Sadah, and Taiz being the worst affected.^[41,43]^ The results also showed increased use of LARCs among women from wealthy households, which is consistent with the literature in Asia.^[16,32]^

Although this study contributes to the literature on the proximate determinants of LARCs’ use in low-resource settings and provides important information on contraceptive use in Yemen, some limitations must be acknowledged when interpreting the findings. First, this was a cross-sectional study involving women of reproductive who had no plans to have children. As a result, associations can only be drawn, and the findings may not apply to women with fertility plans. Second, the participants claimed to use contraception, which the research team could not verify. Consequently, some individuals probably gave positive responses. However, recent research suggests that self-reported information on current contraceptive use by women is accurate because it is connected with nontrivial amounts of inconsistent reporting.^[44]^ Finally, the current study analyzed a secondary dataset, which limited the number of variables that could be investigated to those in the dataset.

## 5. Conclusions

From this study, we learned that the majority of married women in Yemen, who no longer desired children, did not use any form of contraception. We also discovered that the majority of women who used contraception were on SARCs, which are known to be less effective in preventing unwanted pregnancies than LARCs. Several predisposing, enabling, and need factors have been identified that increase women’s likelihood of utilizing LARCs, including higher maternal education, urban domicile, and high- wealth groups. Women in Yemen who are married and want to have fewer children may use LARCs more often if they have more education and are better off financially.

## Acknowledgments

The authors are grateful to ICF International for their approval for using the dataset.

## Author contributions

All authors critically reviewed the first draft, agreed on the final version, agreed on the journal to which the manuscript should be submitted, and agreed to take responsibility and be accountable for the contents of the article.

**Conceptualization:** Michael Boah, Abdul-Nasir Issah, Dalia Hyzam.

**Data curation:** Michael Boah.

**Formal analysis:** Michael Boah, Abdul-Nasir Issah, Isaac Demuyakor.

**Methodology:** Michael Boah, Abdul-Nasir Issah, Dalia Hyzam.

**Writing – original draft:** Dalia Hyzam.

**Writing – review & editing:** Michael Boah, Abdul-Nasir Issah, Isaac Demuyakor.
